# Extramedullary Myelopoiesis in Malaria Depends on Mobilization of Myeloid-Restricted Progenitors by IFN-γ Induced Chemokines

**DOI:** 10.1371/journal.ppat.1003406

**Published:** 2013-06-06

**Authors:** Nikolai N. Belyaev, Judit Biró, Jean Langhorne, Alexandre J. Potocnik

**Affiliations:** 1 Division of Molecular Immunology, MRC National Institute for Medical Research, London, United Kingdom; 2 Division of Parasitology, MRC National Institute for Medical Research, London, United Kingdom; McGill University, Canada

## Abstract

Resolution of a variety of acute bacterial and parasitic infections critically relies on the stimulation of myelopoiesis leading in cases to extramedullary hematopoiesis. Here, we report the isolation of the earliest myeloid-restricted progenitors in acute infection with the rodent malaria parasite, *Plasmodium chabaudi*. The rapid disappearance of these infection-induced myeloid progenitors from the bone marrow (BM) equated with contraction of the functional myeloid potential in that organ. The loss of BM myelopoiesis was not affected by the complete genetic inactivation of toll-like receptor signaling. De-activation of IFN-γ signaling completely abrogated the contraction of BM myeloid progenitors. Radiation chimeras of *Ifngr1*-null and control BM revealed that IFN-γ signaling in an irradiation-resistant stromal compartment was crucial for the loss of early myeloid progenitors. Systemic IFN-γ triggered the secretion of C-C motif ligand chemokines CCL2 and CCL7 leading to the egress of early, myeloid-committed progenitors from the bone marrow mediated by their common receptor CCR2. The mobilization of myeloid progenitors initiated extramedullary myelopoiesis in the spleen in a CCR2-dependent manner resulting in augmented myelopoiesis during acute malaria. Consistent with the lack of splenic myelopoiesis in the absence of CCR2 we observed a significant persistence of parasitemia in malaria infected CCR2-deficient hosts. Our findings reveal how the activated immune system mobilizes early myeloid progenitors out of the BM thereby transiently establishing myelopoiesis in the spleen in order to contain and resolve the infection locally.

## Author Summary

Malaria in man and in most animal models is accompanied by splenomegaly. At the same time, the spleen is the main organ for the control resolution of the parasitemia. This process initially depends mostly on the innate immune system and requires increased production of myeloid cells. We investigated the number of bone marrow (BM) LIN^−^ cells which includes hematopoietic stem cells and progenitors during infection of mice with *Plasmodium chabaudi* and observed a significant reduction. Using a refined definition for early myeloid-restricted progenitors we could show that the loss of these cells in malaria equated with contraction of BM myelopoiesis. Since absence of IFN-γ receptor on stromal cells was sufficient to block this contraction we investigated the effect of IFN-γ on chemokine secretion. We observed a huge upregulation of CCL2/CCL7 serum levels and an increase in *Ccl2/Ccl7* transcription in the BM at peak parasitemia. Egress from the BM of early myeloid progenitors was critically dependent on the chemokine receptor CCR2. Their mobilization resulted in extramedullary myelopopiesis in the spleen which contributed to the clearance of parasite-infected erythrocytes. Our study defined the molecular signals and interaction of various cell types leading to the establishment of splenic myelopoiesis in a mouse model of malaria.

## Introduction

Myeloid cells play essential roles in the control of infection such as that caused by malaria parasite, *Plasmodium*. Although the ultimate control of blood-stage malaria in mice depends on an adaptive immune system [1], the removal of parasite-infected red blood cells requires an efficient early response of innate cells such as monocytes, macrophages and dendritic cells [2]. The production and mobilization of downstream myeloid precursors and in particular “inflammatory monocytes” [3] during experimental malaria and other infections [4], [5], [6] is well documented. However, despite their obvious importance for the continuous maintenance and replenishment of the innate immune system, information on the functional and phenotypical composition of more upstream, early myeloid-restricted progenitors, and changes in the composition and compartmentalization of hematopoietic stem cells (HSCs) and hematopoietic progenitor cells (HPCs) during acute and chronic infection is lacking.

Under homeostatic conditions HSCs and HSPs are compartmentalized in the bone marrow (BM) and replenish the hematopoietic system constitutively. In particular, innate immune cells are produced constantly at high numbers and are released into the circulation in steady state and in response to infection or inflammation. In steady-state hematopoiesis, both HSCs and HPCs reside mostly in specialized niches in the BM cavity that control their survival, proliferation, self-renewal, and differentiation [7], but it is not known whether and how these progenitor cells are mobilized in response to infection or to the accompanying inflammation.

To address these questions in malaria, we have used the mouse model of *Plasmodium chabaudi AS*, where we have previously described the emergence of atypical progenitor cells in BM [8], and the mobilization of “inflammatory monocytes” [9], which participate in parasite control. The resolution of acute parasitemia in several malaria models is positively correlated with quality and quantity of splenic myelopoiesis. In both humans and mice, macrophage hyperplasia is associated with splenomegaly [10], and disruption of the splenic microarchitecture has been reported in lethal cases of infection with *P. falciparum* in humans [11] as well as in mouse malaria models [12], [13]. Furthermore, resolution of the acute parasitemia could be positively correlated with quality and quantity of splenic myelopoiesis during infection [14], [15] and provides strong evidence for the impact of infection on the homeostasis of hematopoiesis and the compartmentalization of hematopoietic development.

Here, we report a refined resolution of HPCs that allows monitoring of early myeloid-restricted progenitor subsets during infection with *P. chabaudi*. The strong contraction in the numbers of myeloid committed progenitors correlated with the functional loss of early myelopoiesis in the BM. We have established that this process is critically dependent on interferon γ (IFN-γ) signaling resulting in the mobilization of CCR2-expressing HPCs in a mechanism involving secretion of IFN-γ induced chemokines by a non-hematopoietic BM compartment. In the absence of CCR2 this mobilization did not occur accompanied by an obvious lack of extramedullary myelopoiesis in the spleen. These data demonstrate that the pro-inflammatory cytokine IFN-γ not only directly shapes early hematopoiesis by acting on HSCs and HPCs during infection, but also mediates the recruitment of these cells to extramedullary sites. Our findings link the dynamic re-modeling of myelopoiesis, which represents a characteristic element of mouse malaria models, to the induction of extramedullary hematopoiesis which actively contributes to the effective initial control of the parasite in the host by the innate immune system.

## Results

### Loss of lineage-negative BM cells and Sca-1 upregulation in *P. chabaudi* malaria are both dependent on IFN-γ signaling

**Figure 1:**
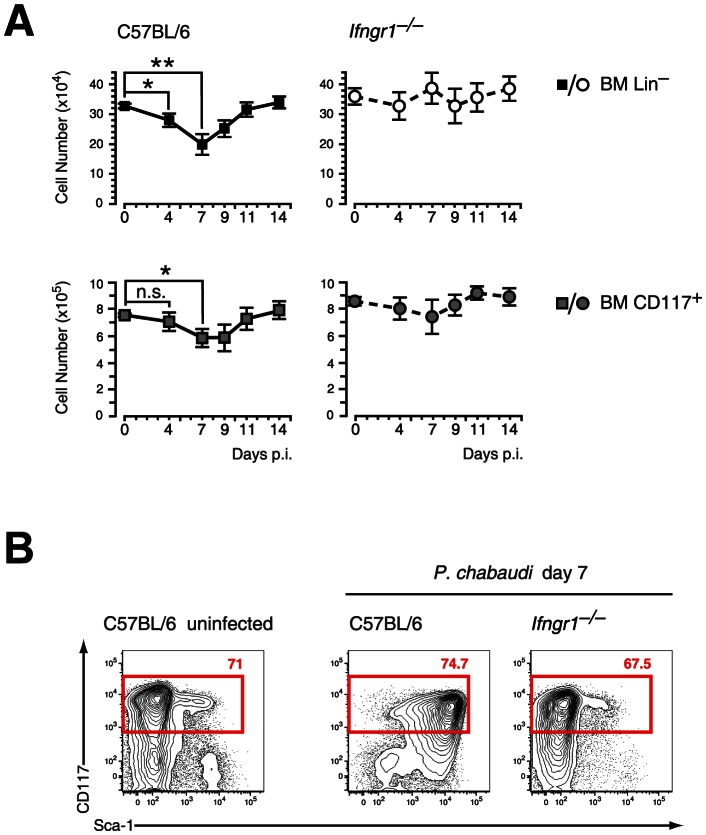
Contraction of lineage negative cell and Sca-1 upregulation in malaria are dependent on IFN-γ signaling. **A.** Reduction of LIN^−^ and c-Kit (CD117) positive cells in the BM during infection of C57BL/6 mice with *P. chabaudi*. Significant contraction of absolute numbers was recorded at day 4 and day 7 after infection for LIN^−^ cells and at day 7 for c-Kit^+^ cells. *Ifngr1*-null mice did not undergo any significant loss of LIN^−^ or c-Kit^+^ BM cells during acute malaria. Data represent mean ± SEM of cell number per femur/pair obtained from 15 infection experiments (C57BL/6) or four experiments (*Ifngr1*-null mice) each with 4–5 mice per group (*:*P*≤0.05; **: *P*≤0.01, Mann-Whitney U-test). **B.** LIN^−^ BM cells from uninfected controls and malaria-infected animals at day 7 of infection with *P. chabaudi* were co-stained with c-Kit and Sca-1. Of note is the lack of Sca-1 upregulation on *Ifngr1*­null cells during acute malaria. Data are representative for 19 infection experiments (C57BL/6) or four experiments (*Ifngr1*-null mice) each with numbers in individual plots indicating the frequency (in %) of the respective population.

Infection of C57BL/6 mice with the malaria parasite *P. chabaudi* results in an acute systemic infection characterized by peak parasitemia at day 7 of infection (**Figure S1**) and subsequent severe anemia. Parallel to the acute infection the absolute number of LIN^−^ BM cells, mainly consisting of hematopoietic progenitors and precursors, were significantly decreased in infected wild type mice resulting in the lowest number of LIN^−^ cells at peak parasitemia (**Figure 1A**). Similarly we observed a significant reduction in the number of c-Kit^+^ (CD117) BM cells. This infection-induced process was accompanied by changes in the phenotype of these LIN^−^ cells. HSCs and multipotent progenitors are characterized in steady state by high expression of c-Kit and Sca-1 [16]. In contrast, HPCs are negative for Sca-1 but still retain high levels of c-Kit at the surface. The HPC subset constitutes the most abundant cell pool within the LIN^−^ pool in the BM. At day 7 of infection with *P. chabaudi* the LIN^−^ compartment showed an upregulation of Sca-1 expression on virtually all c-Kit^hi^ cells (**Figure 1B**). Since Sca-1 is induced by pro-inflammatory cytokines [17], namely IFN-γ, we investigated the cellularity and phenotype of the LIN^−^ compartment during acute malaria in the absence of IFN-γ signaling. Mice deficient for IFN-γ receptor did not show a significant change in the numbers of LIN^−^ or c-Kit^+^ cells in the BM (**Figure 1A**). In addition, expression of c-Kit and Sca-1 remained unchanged on LIN^−^ cells of *Ifngr1*-null mice at day 7 of infection indicating the dependence of Sca-1 induction on intact IFN-γ signaling (**Figure 1B**).

**Figure 2:**
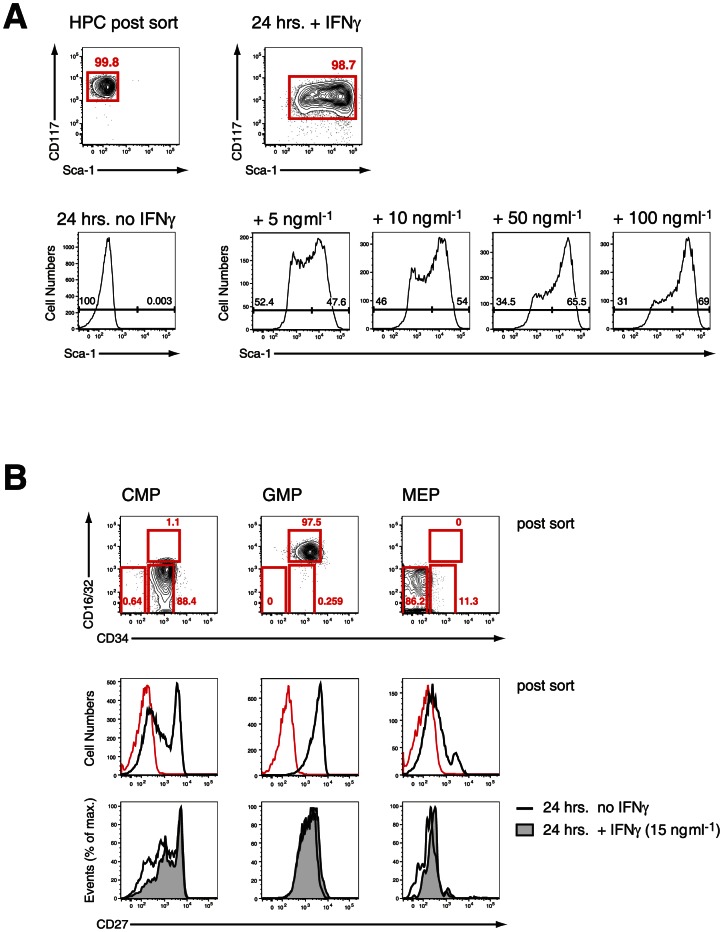
IFN-γ in vitro effectively upregulates Sca-1 on HPCs without affecting expression of CD27. **A.** FACS-purified steady-state HPC were cultured in various concentration of IFN-γ for 24 hrs *in vitro*. Representative histograms from three experiments are depicted. **B.** CMPs, GMP, MEP were FACS-purified from steady state HPCs and stained for expression of CD27 before (post sort, red line shows “fluorescence minus one” FMO control) and after culture with IFN-γ for 24 hrs. Histograms from one of two experiments are shown.

Next we investigated the effect of IFN-γ on FACS-purified HPCs *in vitro*. After short-term culture (24 hrs) virtually all recovered HPCs upregulated Sca-1 to various extents (**Figure 2A** **, upper panel**). There was a biphasic expression of Sca-1 on HPCs, which was dependent on the concentration of exogenous IFN-γ added (**Figure 2A** **, lower panel**). These results clearly demonstrated that IFN-γ signaling *in vivo* and *in vitro* is both necessary and sufficient for the upregulation of Sca-1. Considering that the HPCs under homeostatic conditions are Sca-1 negative the upregulation of this antigen during infection or *in vitro* culture of HPCs with IFN-γ creates a problem for the analysis of stem cells and early progenitor subsets in infection. We therefore tested a panel of surface markers on pre-immune BM progenitor subsets and determined whether their expression was regulated by IFN-γ *in vitro*. We isolated HPCs and further subdivided this compartment into the common myeloid progenitor (CMP, CD34^+^ CD16/32^lo/neg^), the granulocyte monocyte progenitor (GMP, CD34^+^ CD16/32^hi^) and the megakaryocyte erythroid progenitor (MEP, CD34^−^ CD16/32^lo/neg^), with the CMP containing a common myelo-erythroid progenitor pool [18]. Among the markers analyzed, CD27 is reportedly expressed on all myeloid BM progenitors [19] and was present *ex vivo* on all GMPs, a large fraction of CMPs, but virtually no MEPs (**Figure 2B** **, middle panel**). After short-term culture with IFN-γ we observed no change of CD27 expression on GMPs and most CMPs but a selective disappearance of CD27^−^ cells from the CMP pool (**Figure 2B** **, lower panel**). The common progenitor for megakaryocytes and erythroid cells (MEP) was particular sensitive to IFN-γ treatment and underwent partially apoptosis, which also might explain the reduction of CD27^−^ cells in the CMP cultures. Taken together, these results suggest that CD27 expression on myeloid progenitors is not modulated by IFN-γ *in vitro* and reliably distinguished myeloid progenitors from MEPs.

### Defining early myeloid restricted progenitors during *P. chabaudi* infection

**Figure 3:**
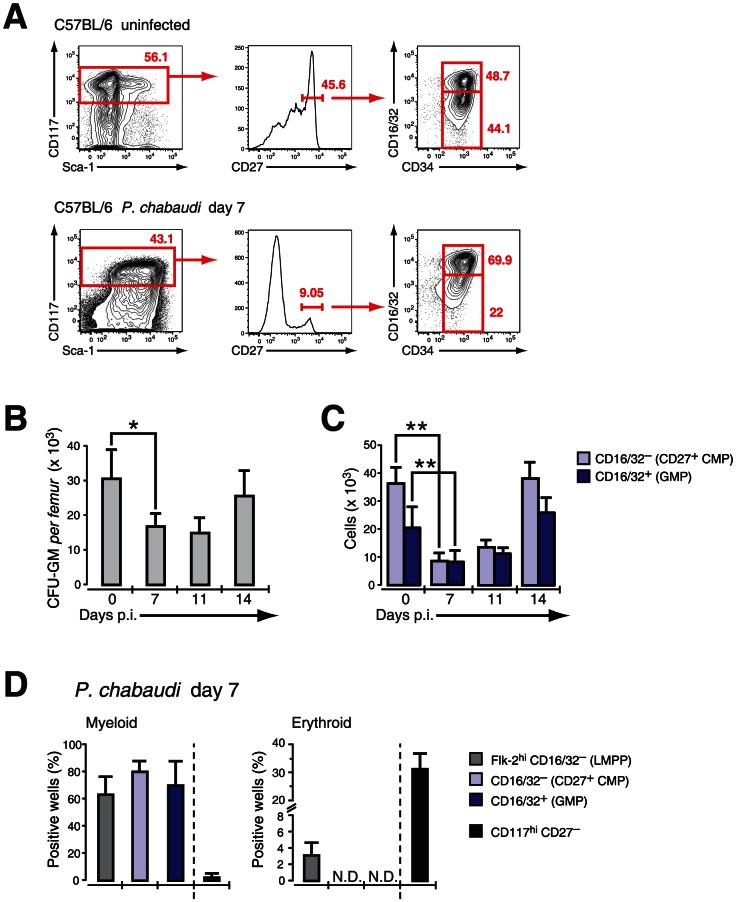
Effect of acute infection with *P. chabaudi* on myeloid progenitor cells in the bone marrow. Infection of C57BL/6 female mice (6–8 weeks) with *P. chabaudi* resulted in a transient decrease of myeloid lineage progenitors in the bone marrow (BM) during acute infection. **A.** BM cells from uninfected controls and malaria-infected animals at day 7 of infection with *P. chabaudi* were co-stained with antibodies against lineage markers, Sca-1, CD117 (c-Kit), CD27, CD34, CD16/32 (FcγRII/III) and separated as indicated. Doublets were electronically excluded prior to analysis. Data are representative for 19 infection experiments with numbers in individual plots indicating the frequency (in %) of the respective population. **B.** Analysis of granulocyte-monocyte colony forming units in semi-solid media from total BM during acute malaria. Data are from two experiments with 4–5 BM samples per experiment and shown as mean ± SEM of total number of CFU-GM per femur (*: *P*≤0.05, two-tailed Student's *t*-test). **C.** Absolute cell numbers of the CD27^+^ CMP (light blue bars) and the GMP (blue bars) progenitor populations during the course of a malaria infection. Data represent mean ± SEM of cell number per femur/pair obtained from 19 infection experiments with 4–5 mice per group (**: *P*≤0.01, Mann-Whitney U-test). **D.** Single cell *in vitro* cultures that support myeloid or erythroid differentiation of FACS purified hematopoietic BM progenitors obtained at day 7 after infection with *P. chabaudi*. Data are shown as mean ± SEM of individual positive wells (in %) of three infection experiments with 5 mice per subset (N.D. = not detected).

As CD27 did not show any qualitative or quantitative change on myeloid progenitors but also no induction on erythroid progenitors *in vitro*, we used expression of this molecule in mice infected with *P. chabaudi* to resolve the different progenitors in the LIN^−^ BM compartment. Cells expressing high levels of c-Kit (CD117) were further gated on CD27 and subdivided in “CD27^+^ CMPs” (54.3±7.3% of LIN^−^c-Kit^+^ CD27^+^ cells), lacking FcγR II/III (CD16/32), and the more advanced “GMP” (42.4±9.4% of LIN^−^c-Kit^+^CD27^+^ cells) (**Figure 3A**). The relative frequency of CD27^+^ cells decreased comparing uninfected and infected samples (**Figure 3A**) affecting disproportionally the early CD27^+^ CMP compartment. We next compared the changes in numbers of early myeloid progenitors with the contraction of functionally defined clonogenic myeloid progenitors in the BM during infection. In acute *P. chabaudi* infection the number of functionally defined myeloid progenitors in the BM showed a significant reduction, which coincided with the peak of parasitemia at day 7 after infection (**Figure 3B**). This decrease of functionally defined myeloid progenitors could be directly correlated with the loss of “CD27^+^ CMPs” (36.1±5.8×10^3^ cells uninfected, 8.4±2.9×10^3^ cells day 7 p.i.) and “GMPs” (20.4±7.3×10^3^ cells uninfected, 8.1±3.9×10^3^ cells day 7 p.i.) in the BM during acute infection (**Figure 3C** **,** **Table 1**). At the same time the relative frequency of “CD27^−^ CMPs” (LIN^−^ c-Kit^+^ CD27^−^) increased during infection but their absolute number remained unchanged (**Figure S2**). In the absence of lymphopoiesis during acute infection [8], [20] these data demonstrated that the decrease of the myeloid competence in the BM is the result of a massive reduction in myeloid committed subsets during acute malaria. The significant reduction in the number of BM-resident myeloid committed cells during infection was not caused by apoptosis or changes in the proliferative state of these subsets since both parameters were largely identical with steady-state populations (**Figure S3**). To ensure that the myeloid potential of the infection-induced hematopoietic progenitors was not altered, we tested their myeloid and erythroid potential *in vitro* (**Figure 3D**). The cloning efficiencies in this single cell assay demonstrated highly efficient generation of myeloid colonies by “CD27^+^ CMP” and “GMP” comparable to lymphoid-biased multipotent progenitors (LMPP; LIN^−^Sca-1^+^ c-Kit^hi^ Flk-2^hi^) whereas “CD27^−^ CMP” did not harbor myeloid potential. Conversely only the latter showed the capacity to produce erythroid cells, which was completely absent in the two myeloid subsets. These results demonstrate that the transient reduction of myelopoiesis in BM after infection with *P. chabaudi* is the direct consequence of a loss in the number of myeloid-restricted BM progenitors.

**Table 1:** Compilation of absolute numbers of CMP and GMP of bone marrow cells in steady state and at day 7 after infection with *Plasmodium chabaudi* in C57BL/6 mice and various mutant mouse strains.

		BM cell number per femur/pair (×10^3^)	
		CMP	GMP
**C57BL/6**	uninfected	36.1±5.8	20.4±7.3
	Day 7 post infection	8.4±2.9	8.1±3.9
	Ratio uninfected∶infected	4.3	2.5
	% at day 7 post infection	23%	39%
***Ifngr1^−/−^***	uninfected	37.2±11.8	38.0±15.5
	Day 7 post infection	30.7±11.0	51.3±12.8
	Ratio uninfected∶infected	1.2	0.74
	% at day 7 post infection	83%	135%
***Ccr2^−/−^***	uninfected	60.6±5.0	41.8±8.4
	Day 7 post infection	50.7±7.2	59.3±6.5
	Ratio uninfected∶infected	1.2	0.7
	% at day 7 post infection	83%	142%
***Myd88^−/−^ Trif^−/−^***	uninfected	60.9±5.6	40.9±1.9
	Day 7 post infection	32.9±6.5	26.4±5.8
	Ratio uninfected∶infected	1.9	1.5
	% at day 7 post infection	54%	65%

**CMP:** LIN^−^ c-Kit^+^ CD27^+^ CD34^+^ CD16/32^−^.

**GMP:** LIN^−^ c-Kit^+^ CD27^+^ CD34^+^ CD16/32^+^.

### Suppression of BM myelopoiesis during infection is critically dependent on IFN-γ signaling in irradiation-resistant cells

**Figure 4:**
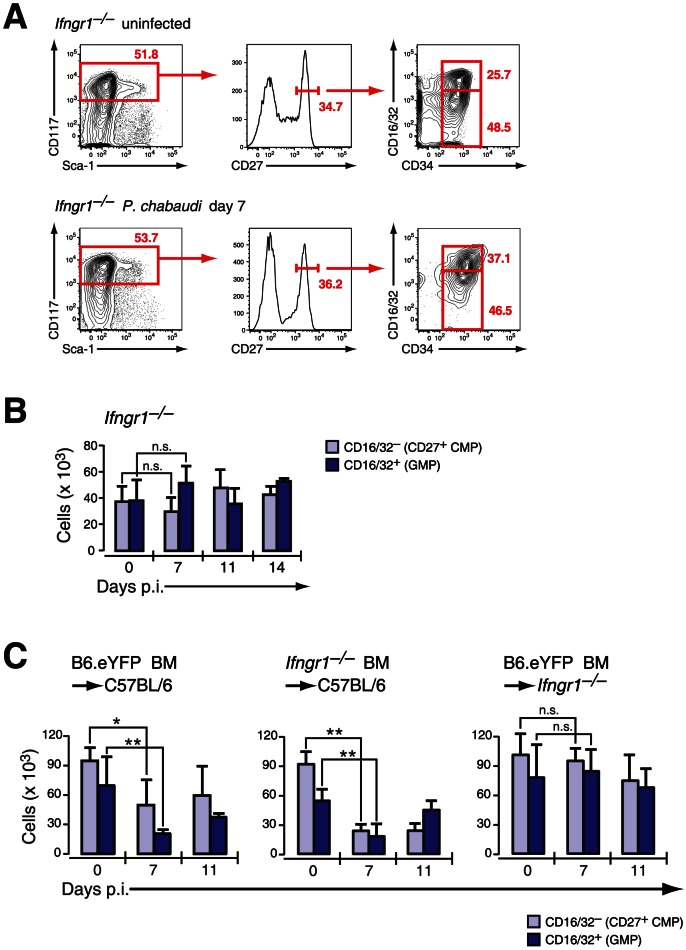
Infection-induced decrease of early myeloid progenitors is critically dependent on IFN-γ signaling. **A.** Phenotype of LIN^−^ BM cells in *Ifngr1*-null mice uninfected and at day 7 after infection with *P. chabaudi* stained for indicated marker. Representative FACS plots of four experiments with each 5 mice per group are shown. **B.** Absolute numbers (per femur/pair) for *Ifngr1*-null LIN^−^ c-Kit^hi^ CD27^+^ cells are shown as mean ± SEM per individual subsets obtained form three experiments with 5–6 mice per group (n.s.: *P*>0.05, Mann-Whitney U-test). **C.** Absolute numbers of myeloid progenitors in radiation chimeras (Hosts: C57BL/6, left and middle; *Ifngr1*-null, right chart) reconstituted either with B6.Rosa26^eYFP^ or *Ifngr1*-null and 8–9 weeks after transplantation infected with *P. chabaudi*. Two different sets of chimeras were infected and 5–9 animals per time point each were analyzed. Data represent mean ± SEM of cell number per femur/pair (*: *P*≤0.05; **: *P*≤0.01, Mann-Whitney U-test).

We next focused on the signals underlying the transient contraction of BM myelopoiesis. Since toll-like receptors (TLRs) are expressed on HSCs and HPCs [21] we first investigated the composition of BM hematopoietic progenitors during acute infection with *P. chabaudi* in mice deficient for MyD88 and TRIF adaptor proteins. *Myd88/Trif*-null mice exhibited similar changes of their phenotypic composition in the LIN^−^ compartment as wild type controls during acute malaria (**Figure S4A**) accompanied by splenomegaly comparable to wild type controls (spleen weight controls uninfected: 47.8±12.4 mg, controls day 7 after infection: 234±63.2 mg; *Myd88/Trif*-null uninfected 38.1±19.7 mg, *Myd88/Trif*-null day 7 after infection: 207.2±89.1 mg). The complete deactivation of TLR-signaling did not alter the early contraction of the absolute numbers of “CD27^+^ CMPs” (60.9±5.6×10^3^ cells uninfected, 32.9±6.5×10^3^ cells day 7 p.i.) and “GMPs” (40.9±1.9×10^3^ cells uninfected, 26.4±5.8×10^3^ cells day 7 p.i.) (**Figure S4B,** **Table 1**). Given the impact of IFN-γ for the modulation of surface antigens in HSCs and HPCs and its importance in the control of parasitemia in malaria we investigated the role of this cytokine in the contraction of BM myelopoiesis. As expected no major alteration in the composition of pre-immune and infection-induced early myeloid subsets was recorded in the absence of IFN-γ signaling for “CD27^+^ CMPs” (39.3±12.3% of LIN^−^c-Kit^+^CD27^+^ cells) or “GMPs” (34.3±13.0% of LIN^−^c-Kit^+^CD27^+^ cells) (**Figure 4A**). Accordingly, no reduction in the number of “CD27^+^ CMPs” (37.2±11.8×10^3^ cells uninfected, 30.7±11.0×10^3^ cells day 7 p.i.) or “GMPs” (38.0±15.5×10^3^ cells uninfected, 51.3±12.8×10^3^ cells day 7 p.i.) was observed in the BM after malaria infection when compared to their pre-immune numbers (**Figure 4B** **,** **Table 1**). In addition, the myeloid competence of multipotent progenitors (LMPP) and myeloid restricted subsets (CD27^+^ CMP and GMP) was not altered in the absence of IFN-γ receptor before and during acute malaria, excluding a change of developmental potential in response to lack of IFN-γ signaling in steady-state or after infection (**Figure S5**).

In order to determine whether the reduction of myeloid progenitors was a direct result of IFN-γ acting on the hematopoietic cells themselves we generated radiation chimeras of C57BL/6 recipients with either IFN-γ receptor-positive (B6.Rosa26^eYFP^) or IFN-γ receptor-negative (*Ifngr1*-null) BM. In both cases an acute *P. chabaudi* infection resulted in transient decrease of “CD27^+^ CMP” and “GMP” numbers in the BM (**Figure 4C**) indicating that IFN-γ signaling in the hematopoietic compartment was dispensable for the reduction in early myeloid progenitors. In contrast, *Ifngr1*-null recipients receiving BM cells proficient in IFN-γ signaling (B6.Rosa26^eYFP^) did not exhibit any decreased number of early BM myeloid progenitors. Therefore the quantitative reduction of BM myeloid progenitor pools was dependent on intact IFN-γ signaling in an irradiation-resistant cellular compartment and independent from direct or indirect signals through TLRs.

### Absent contraction of myeloid progenitors during acute malaria in *Ccr2*-null animals in the presence of intact IFN-γ signaling

**Figure 5:**
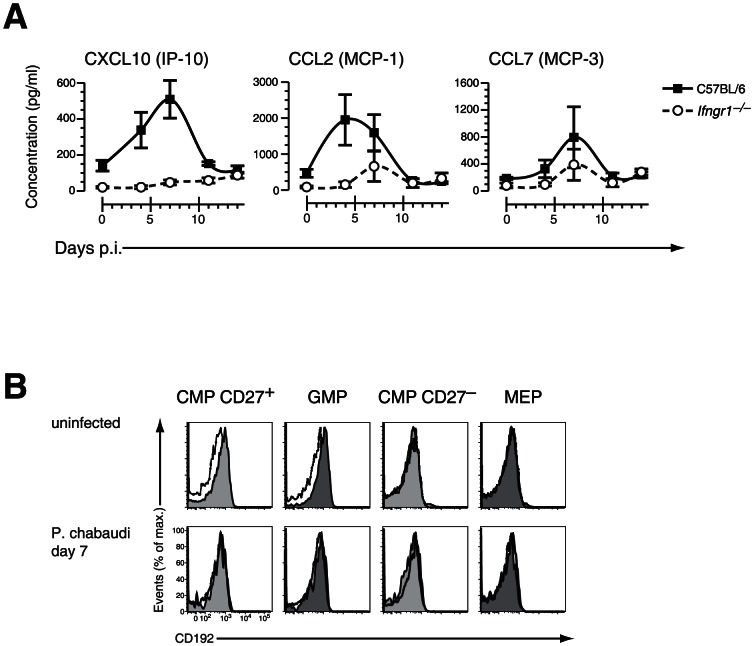
Changes in chemokines and chemokine receptor expression during acute malaria. **A.** Serum concentration of IFN-γ induced chemokines in C57BL/6 and *Ifngr1*-deficient mice infected with *P. chabaudi*. Data are shown as mean ± SEM of serum concentration of respective chemokine in three infection experiments with 5–6 mice per each experimental group. **B.** Cell surface expression of CD192 (CCR2) on myelo-erythroid progenitors in the BM of mice infected with *P. chabaudi* day 7 (lower part) or before infection. All data are representative of two independent experiments with 4–5 mice per group each. Black line represents signals in *Ccr2^−/−^* animals for comparison.

To dissect further the molecular mechanism resulting in a depletion of early stages of BM myeloid progenitors we analyzed the pattern of IFN-γ-induced cytokines and chemokines during acute infection with *P. chabaudi* in wild-type and *Ifngr1*-null mice. After infection of wild-type mice with *P. chabaudi* the Stat1-dependent chemokines CXCL10 (IP-10), CCL2 (MCP-1) and CCL7 (MCP-3) reached relative serum peak levels by day 7 after infection with the notable exception of CCL2 which was the most abundant in the circulation already at day 4 (**Figure 5A**). The upregulation of these chemokines was critically dependent on IFN-γ signaling as demonstrated by their virtual absence in *Ifngr1*-null animals during acute malaria. Furthermore the increase in levels of CXCL10 and CCL7 occurred subsequently to the infection-induced upregulation of IFN-γ, since peak levels for this cytokine were reached in C57BL/6 mice 2–3 days earlier (**Figure S6**). Remarkably the levels of serum CCL2 followed closely the respective kinetics for IFN-γ in C57BL/6 wild type mice. *Myd88/Trif*-null mice exhibited a pattern of secretion of IFN-γ (**Figure S6**) as well as IFN-γ induced chemokines mostly identical to C57BL/6 controls with a peak of CCL2 at day 4 of infection (**Figure S7**). The common receptor for CCL2 and CCL7, C-C chemokine receptor type 2 (CCR2; CD192), was measured on the surface of BM HPCs by flow cytometry. In uninfected mice both CD27^+^ CMP and GMPs stained dimly positive for CCR2 whereas CD27^−^ CMPs and MEPs were negative (**Figure 5B** **, upper panel**). After infection with *P. chabaudi* the strongly reduced compartment of BM “CD27^+^ CMPs” and “GMPs” lacked detectable surface levels of CCR2 (**Figure 5B** **, lower panel**) suggesting that residual myeloid progenitors were unable to egress from the BM via this chemokine-signaling pathway. Based on these observations we hypothesized that stroma-derived CCL2/CCL7 by binding to CCR2 could be the main stimuli for the mobilization of myeloid progenitors from the BM explaining their strongly reduced presence in this compartment.

**Figure 6:**
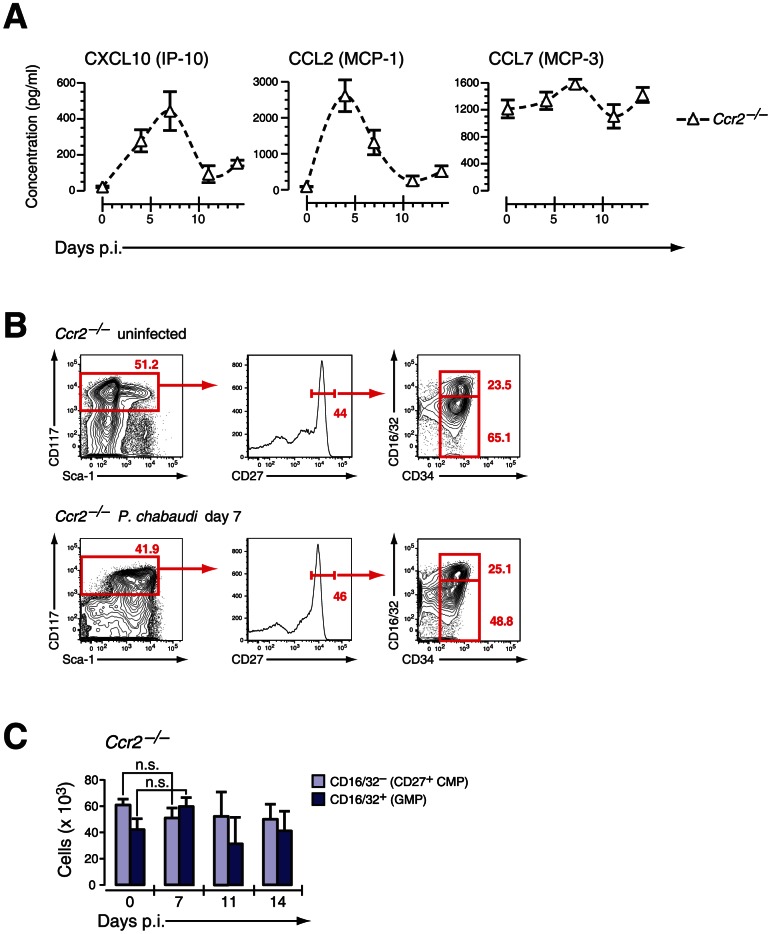
*Ccr2*-null mice induce IFN-γ-dependent chemokines but do not mobilize early myeloid progenitors. **A.** Serum concentration of IFN-γ induced chemokines in *Ccr2*-null mice infected with *P. chabaudi*. Data are shown as mean ± SEM of serum concentration of respective chemokine in three infection experiments with 4–5 mice per each experimental group. **B.** Phenotype of LIN^−^ BM cells in *Ccr2*-null mice uninfected and at day 7 after infection with *P. chabaudi* stained for indicated marker. **C.** Absolute numbers (per femur/pair) for *Ccr2*-null LIN^−^ c-Kit^hi^ CD27^+^ cells are shown as mean ± SEM per individual subsets during infection with *P. chabaudi*.

We therefore next investigated the consequences of CCR2 deficiency on BM hematopoiesis during acute infection with *P. chabaudi*. *Ccr2*-null mice exhibited a similar upregulation of IFN-γ plasma levels within the first four days after infection compared to controls demonstrating a virtually intact IFN-γ response of CCR2 deficient hosts (**Figure S6**). In malaria, both CXCL10 and CCL2 serum protein levels increased reaching peak levels between day 4 and 7 after infection whereas CCL7 was constitutively deregulated in *Ccr2*-deficient animals (**Figure 6A**). The similar increase of IFN-γ levels in *Ccr2*-null animals and controls after infection with *P. chabaudi* was reflected by an analogous upregulation of Sca-1 in BM *Ccr2*-null LIN^−^ cells and the amalgamation of HSC and HPC compartments (**Figure 6B** **, lower panel**). Despite this transformation, absence of CCR2 completely abolished the disappearance of BM myeloid progenitors, for both “CD27^+^ CMPs” (60.6±5.0×10^3^ cells uninfected, 50.7±7.2×10^3^ cells day 7 p.i.) and “GMPs” (41.8±8.4×10^3^ cells uninfected, 59.3±6.5×10^3^ cells day 7 p.i.), during acute malaria (**Figure 6C** **,** **Table 1**). In *Ccr2*-null mice we found a clear dissociation between IFN-γ signaling in the BM as evident by the infection-induced changes in their phenotype but without any contraction in the numbers of myeloid-restricted early progenitors. Since absence of CCR2 did not affect the kinetics of the amount of serum IFN-γ and CCL2, this observation is highly suggestive for a direct engagement of CCR2 leading to the loss of myeloid progenitors.

### CCR2-dependent mobilization of BM progenitors during infection results in the establishment of splenic myelopoiesis

**Figure 7:**
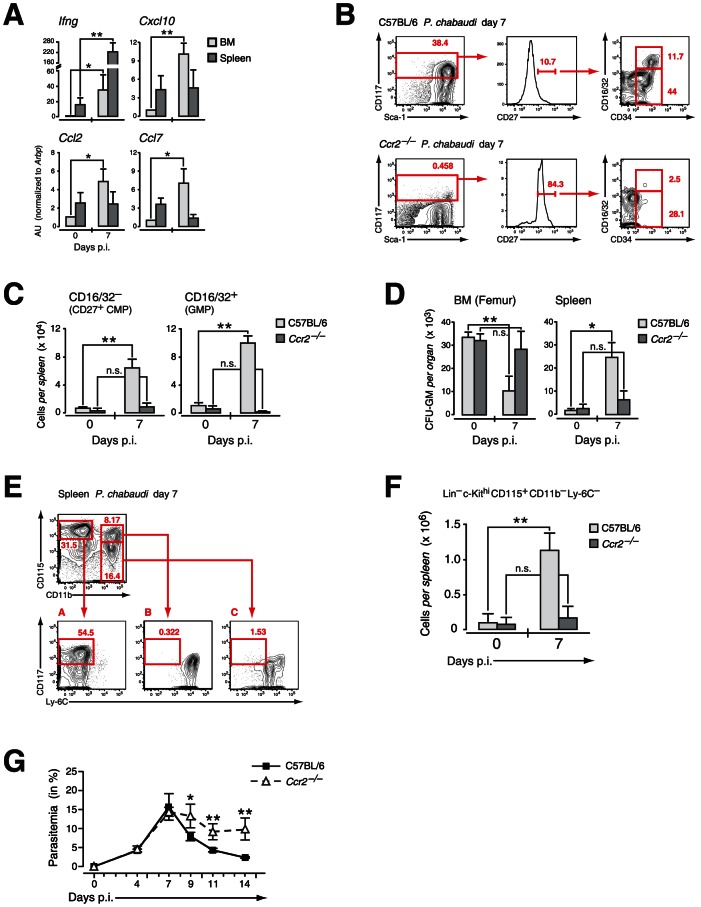
Extramedullary myelopoiesis in malaria is established by mobilizing CCR2^+^ myeloid-restricted BM progenitors. **A.** Expression of message for *Ifng* and IFN-γ induced chemokines in organ samples from pre-immune and mice infected with *P. chabaudi* at day 7. Three to four individual organ samples each were obtained from two independent experiments and analyzed by RT-PCR. **B.** Phenotype of LIN^−^ cells in the spleen of C57BL/6 and *Ccr2*-null mice at day 7 after infection with *P. chabaudi* stained for indicated marker. Results in **B.–E.** are obtained form three infection experiments with 4–5 mice per group each. **C.** Absolute numbers of LIN^−^ c-Kit^hi^ CD27^+^ CD16/32^−^ (CD27^+^ CMPs) and CD16/32^+^ (GMPs) cells in the spleen of C57BL/6 and *Ccr2*-null animals in steady state or at day 7 after infection with *P. chabaudi* as quantified by flow cytometry. Results are shown as mean ± SEM from three independent experiments with 4–5 animals per group (**: *P*≤0.01, Mann-Whitney U-test). **D.** Changes in the absolute number of clonogenic myeloid progenitors (CFU-GM) in the BM and the spleen in uninfected mice and at day 7 after infection with *P. chabaudi*. Data for *Ccr2*-null mice and controls were obtained in three experiments with 4–5 samples per experiment and shown as mean ± SEM of total number of CFU-GM per organ (*: *P*≤0.05, **: *P*≤0.01, two-tailed Student's *t*-test). **E.** Phenotype of intrasplenic myeloid precursors during acute infection with *P. chabaudi*. Lineage-negative (Ter-119, CD3, CD11c, CD19, NK1.1) cells were stained for CD11b versus CD115 (M-CSF receptor) allowing the resolution of early myeloid precursors as c-Kit^hi^ Ly-6C^−^. **F.** Absolute numbers of early myeloid precursors (LIN^−^ c-Kit^hi^ CD115^+^ CD11b^−^ Ly-6C^−^) in the spleen of uninfected mice and at day 7 after infection. Representative staining and splenic cellularity were obtained from three independent infection experiments with 3–5 animals per group. Data represent mean ± SEM of cell number per spleen (**: *P*≤0.01, Mann-Whitney U-test). **G.** Course of malaria infection in C57BL/6 and *Ccr2*-null mice inoculated with blood stages of the malaria parasite *P. chabaudi*. Parasitemia was counted on the indicated days. Results for four infection experiments with 4–5 animals per time point in each experiment are shown as mean ± SEM (*: *P*≤0.05, **: *P*≤0.01, Mann-Whitney U-test).

The decrease in myeloid-restricted BM progenitors clearly linked IFN-γ signaling to chemokine-induced migration. Mindful that the expression of chemokines and cytokines in the serum does not allow the localization of chemokine/cytokine producing cells, we used quantitative RT-PCR to determine the relative expression of *Ifng*, *CXCL10*, *CCL2* and *CLL7* in spleen versus BM during infection with *P. chabaudi* (**Figure 7A**). To avoid artifacts generated by enzymatic manipulation tissue samples were normalized to their dry-frozen weight and RNA was prepared. Whereas *Ifng* message increased both in spleenic and BM samples between day 0 and 7 of infection the IFN-γ-induced chemokines increased significantly only in day 7 BM preparations. Taking into consideration the weight increase of the spleen at day 7 of infection this equates with a strongly increased transcriptional rate per total spleen. In the BM we observed an induction for *Cxcl10*, *Ccl2* and *Ccl7* transcriptional activity. Since HPC including both CD27^+^ and CD27^−^ subsets did not express message for these genes both in steady state and after infection (data not shown) the increase in chemokine message was most likely due to their induction in stromal and vascular cells. At this time point, coinciding with the most significant decrease in the number of BM progenitors, CCR2 expression was undetectable on BM myeloid progenitors (**Figure 5B**) arguing that a local chemokine gradient was established in the BM.

To determine whether the compartmentalization of early myeloid progenitors during malaria was affected by the infection-induced local upregulation of chemokines we analyzed presence and function of early myeloid-restricted progenitors in the spleen. In wild type hosts the infection-induced mobilization of myeloid progenitors via CCL2/CCR2 resulted in the presence of “CD27^+^ CMPs” and “GMPs” in the spleen (**Figure 7B** **, upper panel**), inversely corresponding to their loss from BM following infection. As suggested by the retention of these cells in the BM in *Ccr2*-null mice, no increase in infection-induced early myeloid progenitor subsets in the spleen was recorded in these mice upon infection with *P. chabaudi* (**Figure 7B** **, lower panel;** **Figure 7C**). Furthermore the number of functionally defined myeloid progenitors in the spleen of CCR2 deficient hosts remained virtually unchanged whereas a significant increase in the number of CFU-GM forming cells was observed in wild type controls infected with *P. chabaudi* (**Figure 7D**). This increase in functionally-defined early myeloid progenitors in the spleen during infection paralleled the contraction of these cells in the BM. The increased presence of myeloid progenitors in the spleen during infection equated with the significantly increased myeloid potential of this organ, suggesting establishment of extramedullary myelopoiesis in the spleen (**Figure 7E**). The quantification of c-Kit^hi^ CD115^+^ myeloid precursors revealed a highly significant increase in this cell type at the peak of parasitemia (**Figure 7F**). In the absence of mobilization of myeloid progenitors in *Ccr2*-null mice and an apparent lack of splenic immigration the number of myeloid precursors was equally not increased (**Figure 7F**). The lack of splenic myelopoiesis resulted in a significantly delayed resolution of parasitemia in CCR2 deficient mice when compared to the control group (**Figure 7G**). Thus release of early myeloid progenitors from the BM in acute malaria goes hand in hand with the establishment of extramedullary myelopoiesis thereby contributing ultimately to the resolution of infection.

## Discussion

Despite large progress in the understanding of the developmental pathways and molecular mechanism of steady-state hematopoiesis relatively little is known about the effects of an activated immune system on these biological processes. Many acute and chronic infections result in fundamental changes regarding the production rate of particular hematopoietic lineages and the composition of HSCs and early HPCs (reviewed in [22], [23]). These alterations include a general cessation of lymphopoiesis in the BM [20], [24] and a virtual disappearance of common lymphoid progenitors [8]. The contraction of BM lymphopoiesis in most infectious and noninfectious inflammations is accompanied by expanded myelo- and granulopoiesis [25], [26] providing further evidence for the dynamic reaction of hematopoiesis to exogenous stimuli.

During systemic infection the identification of HSCs and HPCs becomes substantially more complex due to the upregulation of the canonical stem cell marker Sca-1 on HPCs and the resulting phenotypic amalgamation of HSC and HPC compartments documented in a variety of experimental conditions [8], [26], [27], [28]. Acute inflammation associated with *P. chabaudi* as described here, as well as with murine cytomegalovirus [29], and the intracellular bacteria *Ehrlichia muris* [26] results in significant contraction of functionally-defined monocytic and granulocytic progenitors (CFU-GM).

Here we have refined the phenotype-based isolation of early myeloid subsets in the HPC compartment by co-staining with CD27, a member of the TNF-receptor superfamily present on a subset of HSCs [30] and all LMPPs, which did not exhibit any regulation of its surface amount during infection. Based on the strict correlation between CD27 expression and myeloid potential in HPCs we could align the infection-induced decrease of functionally-defined myeloid BM progenitors with the reduction in numbers of phenotypically-defined early myeloid progenitors during infection with *P. chabaudi*. Together with the absence of apoptosis of these subsets *in situ* during infection these observation argue against a cell-intrinsic, infection-induced alteration of their developmental competence.

Myelosuppression in the BM during or following acute infection is not exclusive to malaria. One attractive explanation for the infection-related myelosuppression could be inhibitory signaling via pattern recognition receptors (PPRs) namely TLRs in the BM. The signaling via TLRs expressed on HSCs and HPCs has been shown to interfere with hematopoiesis by directly stimulating HSCs to undergo preferentially myelopoiesis [21] or, more selectively, to induce rapid terminal differentiation of CMPs [31]. In addition TLR-signaling has been implicated in the preferential expansion of monocyte precursors in the BM during bacterial infection [32], and signaling via TLRs contributes to the pathology of malaria both in human and mice (reviewed in [1]). However, we describe here that complete genetic ablation of TLR-signaling did result in a significant decrease of the absolute number of CD27^+^ CMPs and GMPs during acute infection. The ratio of steady state, uninfected precursors to infected precursors indicated that the mobilization of these subsets does not depend critically on the presence of TLR-signaling. In addition we also observed splenomegaly comparable with the wild type controls. We also demonstrated that the combined absence of MyD88 and TRIF did not interfere with the upregulation of Sca-1 on HSCs and HPCs as well as the induction of IFN-γ chemokines in *Myd88/Trif*-null mice. In fact, the modulation of Sca-1 is a common denominator between several infection models, even present in the absence of MyD88 or interferon type I signaling [33]. Furthermore it is thought that the parasite is recognized by several PRRs, some of which are still to be identified and some of which trigger innate responses independently of MyD88 [1]. The genetic dissection of the role of non-TLR PPRs will be highly demanding considering the broadly expression of candidates like mannose receptor C type 2, C-type lectin 2 and sialoadhesin. So far, polymorphisms studies in human show only weak associations with various aspects of severe malaria, which supports the idea that many PRRs and pathways may be acting in concert to activate the innate immune system and bring about a dysregulated cytokine response.

In malaria the infection-induced changes of BM hematopoiesis were completely blocked by lack of IFN-γ signaling without affecting the developmental potential of myeloid progenitors during *P chabaudi* infection. In contrast to the induction of an atypical population of infection-induced myelo-lymphoid progenitors, which we showed to be critically dependent on the presence of IFN-γ receptor on HSCs/HPCs for their emergence [8], the effect of IFN-γ on the contraction of BM myelopoiesis was mediated by an irradiation-insensitive cellular compartment. In HSCs/HPCs the cytokine engages directly the IFN-γ receptor complex and activates a complex of signals ultimately leading to the induction of a particular infection-induced progenitor population. The biology of IFN-γ for the homeostasis of early hematopoietic compartments is poorly understood but aspects as the duration and the effective signal strength in the respective developmental niche are certainly important elements in understanding its particular effect. The mobilization of early myeloid-committed progenitors on the other hand is not dependent on IFN-γ signaling in hematopoietic cells. Other cell types in the BM which express IFN-γ receptors are endothelial and stromal cells. Among the main target genes of IFN-γ in these cells are the prototypic IFN-γ response chemokine CXCL10 (IP-10) but also CCL2 (MCP-1) and CCL7 (MCP-2). The systemic presence of CCL2 and CCL7 in malaria, mainly at early time points in infection, was critically dependent on intact IFN-γ signaling and not affected by the absence of TLR-signalling. Considering that baseline expression of CCL2 both in the blood and in the BM was very low we found significant expression of its receptor CCR2 on early myeloid progenitors by flow cytometry in agreement with a recent report [34]. We also established that in CCR2 deficient mice IFN-γ and CCL2 induction by infection with *P. chabaudi* was similar to infected wild type mice whereas CCL7 was constitutively deregulated. In the absence of the chemokine receptor CCR2 the numbers of early myeloid-committed progenitors in the BM did not decrease clearly suggesting that the infection-induced contraction in functional BM myelopoiesis was a direct consequence of mobilization of CCR2^+^ early myeloid progenitors by IFN-γ induced chemokines. Consistent with the depletion of early myeloid progenitors from the BM during acute malaria we observed a reciprocal increase in extramedullary splenic myelopoiesis. The dislodgment of a significant part of myeloid development to the spleen was completely absent in *Ccr2*-null animals further underscoring the crucial importance of the CCL2/CCR2 axis for the rapid and effective initiation of splenic myelopoiesis. It should be stated that mainly in case of mouse mutant strains rendered deficient for cytokine or chemokine receptors we are dealing with a biological system compensating the absence of the respective element. Hence, there remains the possibility that the lack of progenitor mobilization in *Ifngr1-* and *Ccr2-*null animals during acute malaria is not fully reflecting the situation in wild type hosts, a crucial question for future studies.

Activation of the innate immune system, the local expansion of monocytic cells and their removal of parasite-infected erythrocytes are elements contributing to splenomegaly in malaria [35], which is to some extent indicative of disease severity [36]. One element in the complex function of the spleen during malaria is the clearance of parasites. Recruitment of myeloid progenitors and more advanced monocytic precursors including “inflammatory monocytes” from the BM enables a more efficient removal of parasite-infected erythrocytes which is inline with the observation of prolonged acute parasitemia in *Ccr2*-null hosts [9]. The dislodgement of a substantial proportion of lineage-restricted myeloid progenitors to the spleen and temporary establishment of myelopoiesis is one element contributing to macrophage hyperplasia in the spleen [9]. Similarly, previous reports have documented a delayed resolution of parasitemia in mice deficient for IFN-γ signaling and infected with malaria parasites [37], [38]. The presence of lineage-restricted myeloid progenitors in conjunction with the presence of subsequent stages of monocytic progenitors critically coincided with peak parasitemia. Hence, one might speculatively refer to this infection/inflammation induced mobilization of early hematopoietic cells as an extreme example for the described “immune surveillance” by these subsets under steady-state conditions [39].

The well established role of CCR2 in the directed migration of monocytes to areas of infectious and noninfectious inflammation (reviewed in [40]) has recently been expanded by the notion that stem cells and early progenitor subsets both in the hematopoietic [34] and in the neural system [41], [42] utilized CCR2-dependent chemotaxis for migration and homing. In particular, a small subset of HSCs with a high reconstitutive potential expressed *Ccr2* mRNA albeit CCR2 was not present on the cell surface [43]. Considering the expression of CCR2 on the majority of both CD27^+^ CMPs and GMPs, the IFN-γ dependent mobilization of myeloid progenitors and the subsequent establishment of splenic myelopoiesis in acute malaria represent an early mechanism in the activation of the immune system in response to acute infection. It is also reminiscent of the recently reported CCR2 dependent recruitment of HSCs and HPCs to sites of noninfectious inflammation and their local differentiation to macrophages which assist with tissue regeneration [34]. In both cases the protective function arguably involves local differentiation into specialized myeloid and/or dendritic cell subsets.

Our studies demonstrated that the transcriptional activity of IFN-γ induced chemokines is upregulated during acute malaria in the BM. Most importantly the CCL2 secretion in the BM has been recently attributed to CXC chemokine ligand (CXCL) 12-abundant reticular (CAR) cells [44]. These cells are tightly associated with endothelial cells lining BM sinuses and might represent the non-hematopoietic element establishing a local chemokine gradient. Given that the BM endothelium is fenestrated [45], these CAR cells directly located about the vascular compartment might guide early myeloid progenitors into the bloodstream. Once in the circulation a major proportion of these progenitors will be retained in the spleen during acute malaria. The transcriptional activity of chemokines in the spleen did not exhibit significant changes when normalized to the weight of the organ. Since the spleen undergoes a dramatic change in architecture and size, the local concentration of chemokines in the spleen might therefore also not be homogenous. Whether several local gradients are established facilitating the egress from the BM and homing to the spleen of myeloid progenitors or a general systemic gradient is established during infection are just two possible scenarios. Additional studies will be required to distinguish between these possibilities.

In consequence, the establishment of extramedullary myelopoiesis as a direct response to systemic inflammatory signals illustrates the highly dynamic response of the hematopoietic system. Our findings provide a novel role for the action of the pro-inflammatory cytokine IFN-γ acting via the chemokine receptor CCR2 for the efficient mobilization of hematopoietic progenitors and expansion of hematopoiesis into the spleen during acute infection. This “stress-induced” establishment of extramedullary sites of myeloid development therefore represents an important component of a successful immune response and adds to the arsenal of defense elements of an organism. Deeper understanding of these processes may lead to novel cell-based approaches to control selectively the emigration of specialized subsets of HSC and HPCs and their local differentiation to macrophage effector populations.

## Materials and Methods

### Ethics statement

All animal care and experimental procedures were carried out after review and approval by the MRC National Institute for Medical Research Ethical Review Panel in strict accordance to current UK Home Office regulations, and conducted under the authority of United Kingdom Home Office Project Licence PPL 80/2506 (‘Development and function of innate and adaptive immune responses’, AP) and Project Licence PPL 80/2358 (‘Regulation of immune responses and immunopathology in malaria’, JL). All experiments were designed and conducted to minimize suffering and to comply with the principles of replacement, refinement and reduction.

### Mice and parasites

C57BL/6, B6.SJL-*Ptprc^a^Pep3^b^*/BoyJ (CD45.1), B6.Rosa26^eYFP^ [8], *Ifngr1*- [46] and *Ccr2*-deficient [47] mice were kept under sterile pathogen-free conditions at the animal facility of the MRC National Institute for Medical Research (NIMR). *Myd88/Trif*-double deficient mice were generated by intercrossing *Myd88*-null [48] and *Trif*-null [49] animals. All experimental animals were backcrossed for at least 10 generations onto the C57BL/6 background. Acute blood stage malaria was induced by infecting 6–8 week old mice intraperitoneally with 1×10^5^ *P. chabaudi* parasitized RBCs. For erythrocyte, neutrophil and monocyte counts 5 µl of peripheral blood was collected from the tail vein and analyzed using a VetScan HMII (Abaxis) analyzer. All blood counts were routinely controlled by enumeration of subsets according to standard morphological criteria from Giemsa-stained blood smears. Parasitemias were determined from Giemsa-stained thin blood films with at least 1×10^3^ red blood cells counted per slide. Serum samples were prepared at indicated time points and cytokines and chemokines analyzed by FlowCytomix (Bender Medsystems).

### Flow cytometry and cell isolation

For phenotypic analysis, single cell suspensions were stained with mAbs as indicated. Negative controls were performed using irrelevant isotype-matched control mAbs. Dead cells were excluded from analysis by 7-amino-actinomycin D (7-AAD, Sigma) counterstaining. Antibody clones and suppliers are listed in **Table S1**. Analytical flow cytometry was performed on a FACSCanto II (Becton Dickinson) and data analyzed using FloJo software (Tree Star). For cell sorting, an enrichment of lineage negative cells was performed using biotinylated mAbs against lineage antigens (Ter-119, Gr-1, CD11b, CD11c, NK1.1, CD3, CD8α, CD19, CD41, CD45RB, CD127), followed by streptavidin microbeads (Miltenyi) and a cell separation column (Miltenyi), according to the manufacturer's instructions. Lineage marker positive cells were detected by biotinylated mAbs followed by streptavidin-PE/Alexa610 (Molecular Probes) and together with dead cells (detected by 7-AAD staining; Sigma) and doublets electronically excluded prior to separation on a MoFlo (Dako-Beckman) or a FACSAria II (Becton Dickinson). LMPPs were obtained by sorting for LIN^−^ Sca-1^+^ CD117^hi^ CD135^+^ (LMPPs) cells. CMPs were defined as LIN/CD127/Sca-1^−^ CD117^+^ CD34^+^ CD16/32^lo/−^ then further resolved by CD27. GMPs were LIN/CD127/Sca-1^−^ CD117^+^ CD34^+^ CD16/32^hi^ and MEPs were LIN/CD127/Sca-1^−^ CD117^+^ CD34^−^ CD16/32^lo/−^. Post-sort purity was always ≥98%.

For analytical flow cytometry after electronic elimination of cell doublets and dead cells lineage-negative cells in the BM were defined as Ter-119, Gr-1, CD11b, CD11c, NK1.1, CD3, CD8α, CD19 and CD45RB negative.

### Short term serum-free *in vitro* cultures of hematopoietic progenitors

FACS-purified populations of LIN^−^ c-Kit^hi^ Sca-1^−^ HPCs, CMPs, GMPs, and MEPs were seeded at 1×10^5^ cells in independent wells of a 6-well V-bottom plate (Costar) containing 2 ml X-vivo 15 (BioWhittaker) supplemented with various amount of recombinant murine IFN-γ (R&D Research).

### *In vitro* myeloid and erythroid cell differentiation assays

Semi-solid cultures of freshly isolated, unfractionated BM or spleen cells and sorted progenitor populations from uninfected or *P. chabaudi*-infected mice were prepared using Methocult M3234 (Stem Cell Technologies) supplemented with SCF (50 ng/ml), Flt3L (5 ng/ml), GM-CSF (1 ng/ml), M-CSF (100 ng/ml), TPO (10 ng/ml) (all Peprotech) for myeloid differentiation. Colonies were assessed and counted under an inverted microscope from day 3 to day 12. For confirmation of colony types, colonies were picked with fine-drawn Pasteur pipettes, spun down on slides, and after staining with Giemsa evaluated by light microscopy.

For the evaluation of the myeloid and erythroid potential, pre-sorted progenitor populations were sorted for the second time using a single-cell depositor coupled to a FACSAria II (Becton Dickinson). Single cells were seeded in independent wells of a 96-well V-bottom plate (Costar) containing 50 µl X-vivo 15 (BioWhittaker) supplemented with 10% fetal calf serum (Sigma-Aldrich) and cytokines (all PeproTech). The cytokine cocktail consisted of SCF (50 ng/ml), Flt3L (50 ng/ml), TPO (50 ng/ml), IL-3 (10 ng/ml) and GM-CSF (20 ng/ml) for myeloid conditions and SCF (50 ng/ml), TPO (50 ng/ml), and Erythropoietin (20 ng/ml, R&D Research). After 6 days in culture, individual clones were analyzed as previously published [8].

### Mixed bone marrow chimera and *in vivo* adoptive transfer

Mixed BM chimeras were generated by transplanting lethally-irradiated (2×5 Gy) *Cd45.1* B6.SJL or *Ifngr1*-null mice with at total of 2×10^6^ nucleated BM cells of *Ifngr1*-null or B6.Rosa26^eYFP^ mice. Stable chimeras were infected seven to twelve weeks after transplantation. Cells for adoptive transfer were FACS purified and suspended in endotoxin-free phosphate-buffered-saline (Invitrogen). C57BL/6 mice were sublethally irradiated (2.5 Gy) 4 hours prior to transfer. Recipient mice were injected with 2×10^3^ cells into the tail vein. Mice were killed at indicated time points after transplantation.

### RNA extraction and quantitative RT-PCR

RNA was extracted from freeze-dried specimen of spleen and BM of equal weight using TRI Reagent (Molecular Research Centre) according to the manufacturer's guidelines and reverse transcribed by oligo(dT) priming and Superscript RT II (Invitrogen). The resultant cDNA template, was amplified on an ABI 7900 Sequence Analyzer (Applied Biosystems) for 40 cycles in triplicates. Differences in cDNA input were normalised against *Arbp* expression levels in the same sample and data was analysed by the standard curve method. Primer/probe combinations were purchased from Applied Biosystems (Life Technologies) and the relevant order codes are given in **Table S2**.

### Statistical analysis

*P* values were calculated with two-tailed Student's *t*-test or the Mann-Whitney U-test for data obtained from malaria-infected mice.

## Supporting Information

Figure S1 **Parasitemia in C57BL/6 and** ***Ifngr1*** **-null mice infected with** ***P. chabaudi*** **.** Course of malaria infection in C57BL/6 and *Ifngr1*-null mice inoculated with blood stages of the malaria parasite *P. chabaudi*. Parasitemia was counted on the indicated days. Results for four infection experiments with 4–5 animals per time point in each experiment are shown as mean ± SEM (*: *P*≤0.05, **: *P*≤0.01, Mann-Whitney U-test).
(EPS)

Figure S2 **Relative frequency and absolute number of bone marrow LIN^−^ c-Kit^+^ CD27^−^ cells during acute malaria.** Relative frequency and absolute number of bone marrow LIN^−^ c-Kit^+^ CD27^−^ cells during acute malaria in C57BL/6 mice (9 experiments with 3–5 mice per time point each) and *Ifngr1*-null mice (four experiments with 5 mice per time point each) are shown as mean ± SEM (**: *P*≤0.01, Mann-Whitney U-test).
(EPS)

Figure S3 **Analysis of apoptosis and turnover in bone marrow myeloid progenitor cells during acute malaria.** (**A**) Annexin V binding to LIN^−^ c-Kit^+^ CD27^+^ CD16/32^−^ (CD27^+^ CMP, light blue bars) and CD16/32^+^ (GMP, blue bars) cells was analyzed by flow cytometry in uninfected mice and at day 7 after infection with *P. chabaudi*. (**B**) Naïve mice or mice infected with *P. chabaudi* were administered EdU (5-ethynyl-2′-deoxyuridine) at indicated days and BM collected 2 hrs later. Absolute number of EdU^+^ BM cells was determined in CD27^+^ CMP and GMP subsets. Results are given as mean ± SEM from two independent experiments with 5–6 animals per group.
(EPS)

Figure S4 **Reduction in myeloid-restricted progenitors in the bone marrow is not affected in** ***Myd88/Trif*** **-null mice.** (**A**) Absence of TLR-signaling in *Myd88/Trif*-deficient mice did not block reduction in early myeloid progenitors. Flow cytometry analysis of BM cells stained with indicated marker. (**B**) Absolute numbers (per femur/pair) for LIN^−^ c-Kit^+^ CD27^+^ progenitors are shown as mean ± SEM of individual subsets obtained form three experiments with 5–6 mice per group (*: *P*≤0.05, **: *P*≤0.01, Mann-Whitney U-test).
(EPS)

Figure S5 **Functional potential of bone marrow hematopoietic progenitors in** ***Ifngr1*** **-null mice during acute malaria.** Bone marrow hematopoietic progenitors (LMPP, CD27^+^ CMP, GMP) were FACS-isolated from *Ifngr1*-null mice pre-immune and at day 7 after infection with *P. chabaudi*. Cells were cultured as single cells *in vitro* under myeloid conditions. The myeloid potential of *Ifngr1*-null hematopoietic cells before and during acute infection did not exhibit any significant differences.
(EPS)

Figure S6 **Kinetics of systemic IFN-γ expression during acute infection with** ***P. chabaudi*** **.** Serum concentration of IFN-γ was measured after infection with *P. chabaudi* in C57BL/6 (top, black), *Ifngr1*-null (middle top, blue), *Myd88/Trif*-null (middle bottom, green) and *Ccr2*-null (bottom, red) mice. Serum IFN-γ levels in *Myd88/Trif*-null mice were not determined on day 14 post infection. Data were obtained in at least 3 independent experiments with 4–6 animals per group each and are shown as mean ± SEM.
(EPS)

Figure S7 **Kinetics of systemic IFN-γ induced chemokines in** ***Myd88/Trif*** **mice during acute infection with** ***P chabaudi*** **.** Serum concentration of IFN-γ induced chemokines (CXCL10, top; CCL2, middle; CCL7, bottom) was measured after infection with *P. chabaudi* in *Myd88/Trif*-null mice. Data were obtained from three independent experiments with 5–6 animals per group each and are shown as mean ± SEM.
(EPS)

Table S1 **mAb clones and suppliers.** All monoclonal antibodies utilized in the study are listed according to CD number, alternative name (if available), clone, flurochrome and distributor are listed in **Table S1**.
(DOC)

Table S2 **RT-PCR primer/probes.** Primer/probe combination for real-time PCR are listed in **Table S2**.
(DOC)
