# Supplementary material for: Extramedullary Myelopoiesis in Malaria Depends on Mobilization of Myeloid-Restricted Progenitors by IFN-γ Induced Chemokines
Source: PLoS Pathog. 2013 Jun 6;9(6):e1003406. doi: 10.1371/journal.ppat.1003406 (PMC3675198; doi:10.1371/journal.ppat.1003406)
Supplement: Table S1 — mAb clones and suppliers. All monoclonal antibodies utilized in the study are listed according to CD number, alternative name (if available), clone, flurochrome and distributor are listed in Table S1. (DOC) [file ppat.1003406.s008.doc]

**Table S1: mAb clones and suppliers**

|  |  |  |  |  |
| --- | --- | --- | --- | --- |
| **Antigen** | **Name** | **Clone** | **Fluorochrome** | **Supplier** |
|  |  |  |  |  |
| CD3ε |  | 145-2C11 | APC | eBioscience |
| CD8α |  | 53-6.7 | Biotin | eBioscience |
| CD11b |  | M1/70 | Pacific Blue | eBioscience |
| CD11c |  | N418 | APC-Alexa Fluor 750 | eBioscience |
| CD16/32 | FcγII/III receptor | 93 | PE, PE-Cy7 | eBioscience |
| CD19 |  | 1D3 | Biotin, PE-Cy7 | eBioscience |
| CD27 |  | LG.7F9 | FITC | eBioscience |
| CD34 |  | RAM34 | Alexa Fluor 647 | eBioscience |
| CD45R |  | RA3-6B2 | Biotin | eBioscience |
| CD115 | M‑CSF receptor | 53-2.1 | PE | eBioscience |
| CD117 | c‑Kit | 2B8 | APC-Alexa Fluor 750 | eBioscience |
| CD127 | IL‑7Rα | A7R34 | PE, APC, Biotin | eBioscience |
| CD135 | Flk‑2 | A2F10 | PE | eBioscience |
| CD192 | CCR2 | MC-21 | unlabeled | kindly provided by Dr M. Mack |
| F4/80 |  | BM8 | Biotin | eBioscience |
| Gr‑1 | Ly6G | RB6-8C5 | APC-Alexa Fluor 750 | eBioscience |
| Ly6‑C |  | ER-MP20 | Alexa Fluor 647 | Serotec |
| MHC‑II |  | M5/114.15.2 | PE | eBioscience |
| NK1.1 |  | PK136 | PE | eBioscience |
| Ter‑119 |  | TER‑119 | Biotin | eBioscience |
| Sca‑1 | Ly6A/E | D7 | Pacific Blue | BioLegend |
