# Supplementary material for: Extramedullary Myelopoiesis in Malaria Depends on Mobilization of Myeloid-Restricted Progenitors by IFN-γ Induced Chemokines
Source: PLoS Pathog. 2013 Jun 6;9(6):e1003406. doi: 10.1371/journal.ppat.1003406 (PMC3675198; doi:10.1371/journal.ppat.1003406)
Supplement: Table S2 — RT-PCR primer/probes. Primer/probe combination for real-time PCR are listed in Table S2. (DOC) [file ppat.1003406.s009.doc]

**Table S2: RT-PCR primer/probes**

| **Gene** | **Applied Biosystems Number** |
| --- | --- |
| ***Ifng*** | **Mm01197443_m1** |
| ***Cxcl10*** | **Mm00445235_m1** |
| ***Ccl2*** | **Mm00441242_m1** |
| ***Ccl7*** | **Mm00443113_m1** |
